# Gender differences in homicides. A comparative analysis of 106 fatalities in forensic autopsy data

**DOI:** 10.1007/s12024-024-00847-y

**Published:** 2024-06-06

**Authors:** Cleo Walz, Steffen Eifert, Johanna Görg, Clara-Sophie Schwarz, Christian Steffan, Hauke Brettel, Tanja Germerott

**Affiliations:** 1 Institute of Forensic Medicine, Johannes Gutenberg University Medical Center, Am Pulverturm 3, 55131 Mainz, Germany; 2 Department of Criminology, Criminal Law and Medical Law, Johannes Gutenberg University, Jakob-Welder-Weg 9, 55128 Mainz, Germany

**Keywords:** violent death, gender difference, forensic medicine, prevention, intervention, law enforcement

## Abstract

While most homicides worldwide are committed against men, women and girls are disproportionately affected by domestic violence and its fatal consequences. The Istanbul Convention mandates the prevention of gender-based violence, particularly against women. This study analyzes the characteristics of male and female victims of homicides to provide a comprehensive understanding of the different situations and ways in which men and women are killed. Files of 106 forensic autopsies of homicide victims aged 14 years and older (51.9% males, 48.1% females) from 2012 to 2019 were reviewed. Demographic data, previous history with a focus on the perpetrator-victim relationship, substance influence, type of violence and law enforcement data were recorded. A gender-separated statistical analysis was carried out. Male victims were killed most often outside their own home (63.6%) by acquaintances or friends (54.5%). They were frequently under the influence of substances (56.4%). The perpetrators suffered from addictive diseases in 49.1%, and 52.7% had prior convictions. In both groups, stab/cut injuries were the most common causes of death, but stab/cut and gunshot injuries occurred more frequently in male victims. Female victims were killed most often in their own homes (76.5%) by intimate partners (62.7%). Compared to male victims, violent asphyxiation and blunt force trauma were more common causes of death. Furthermore, there was a higher frequency of multiple injuries (33.3%). The prevalence of a guilty verdict of the perpetrators was nearly equal in both groups. Gender-specific aspects should be considered when investigating homicides and establishing prevention and intervention strategies for interpersonal violence. Protective concepts are easier to establish in the public sphere than in private homes, which is why homicides against women require special attention in society, law enforcement, and legislation. Significant gender differences were found in the circumstances and the perpetrator-victim relationships of homicides. Gender-specific aspects should be taken into account when investigating homicides and developing prevention and intervention strategies. Homicides against women require special attention in society, law enforcement and legislation.

## Introduction

According to the United Nations Office on Drugs and Crime, 457.946 people were killed in homicides worldwide in 2021, 87.995 of the victims were women or girls [1, 2]. In Germany, the rate of female and male victims of completed (not attempted) homicides in 2022 was almost balanced (46.6% male, 53.4% female) [3], so that country-specific influences appear to play a role in these felonies (e.g. organized crime like gang crime). Within homicides, more than 90% of murder suspects worldwide are men [4]. A similar distribution can also be seen in Germany [5].

Most studies comparing differences between male and female victims focus on intimate partner homicides, which affect women more often than men [6–8]. The prevalence rates suggest that homicide is also subject to a gender-specific phenomenon, emphasizing the need to investigate such cases by separating male and female perpetrators and victims. In this way, structural backgrounds of homicides in general and specific gender-related characteristics can be analyzed. The high prevalence rates also highlight the importance of examining potential risk factors to establish prevention strategies.

The German Criminal Code (StGB) recognizes distinct types of homicides. The standard crime of intentional homicide is manslaughter (Sect. 212 StGB). However, if specific objective circumstances or (subjective) intentions of the perpetrator are present during the killing, the act is punishable as murder (Sect. 211 StGB). The law explicitly defines nine circumstances or intentions, referred to as “murder characteristics”, which are associated with a specific degree of reprehensibility and therefore an increased level of injustice (Table 1). As with the distinction between negligent killing and manslaughter, the German Federal Government assumes an increased degree of injustice here, which must lead to an increased sentence. As a result, even one of these characteristics leads to murder and carries the maximum punishment of life imprisonment, as opposed to five to fifteen years for manslaughter. Bodily harm resulting in death is not classified as homicide but as an offense against physical integrity (Sect. 227 StGB). In this case, a perpetrator is punished who intentionally injures the body of another and thereby causes the death of the injured person through negligence [9]. Regardless of the specific crime committed, an individual’s sentence may be reduced or a court may order them to be placed in a psychiatric hospital if their criminal responsibility is limited or excluded (Sects. 20, 21, 63 StGB). In these cases, the offenders are psychologically impaired to such an extent that their capacity for insight and control is limited or suspended.

**Table 1:** Aggravating circumstances, which distinguish murder from manslaughter in Germany

Objective circumstances	(Subjective) intentions
Perfidious	Lust to kill
Cruel	To obtain sexual gratification
By means constituting a public danger	Out of greed
	To facilitate another offence
	To cover up another offence

In the context of the factual distinction regarding which homicide has been committed (e.g. murder or manslaughter), the direct wording of the German Criminal Code does not distinguish between gender-specific differences. It is only in the context of the penalty determination, when the concrete measure of the penalty is determined and, for example, the factual distinction between manslaughter and murder has already been made, that gender-specific differences are to be considered. Particularly, the determination of the penalty (Sect. 46 (2) StGB) should consider motives such as racism, xenophobia, antisemitism, gender-specificity, sexual orientation, and other similar motives or goals of the perpetrator. Incidentally, it was Sect. 46 (2) StGB that was last amended in October 2023 [10]. In this process, gender-specific motives, as well as motives directed against sexual orientation, were included. The Federal Government’s explanatory memorandum on the amendment shows that crimes motivated by the gender or sexual orientation of the victim are becoming increasingly relevant in Germany, so a revision had to be made [11].

Nevertheless, even in the context of the factual distinction between manslaughter and murder, the German Federal Court of Justice recognizes that, among other factors, a gender-specific motivation can lead to the assumption of murder in certain individual cases. In certain cases, the subjective intention of “other base motives” may be present, which constitutes murder and excludes the possibility of punishment for manslaughter alone (Table 1). The German Federal Court of Justice defines a motive as “base” if it is considered to be at the lowest level according to general ethical standards. The court conducts a comprehensive evaluation to determine whether this is the case. This evaluation considers all the circumstances of the offence, the offender’s life circumstances, and their personality [12].

The majority of forensic medicine studies on violence-related deaths focus on specific injury outcomes, such as stab wounds or brain injuries, or the type of violence, such as sharp force or strangulation. However, only a small number of these studies analyze these variables in relation to the gender of the victims [13–15]. Moreover, single studies concerning injury patterns focus exclusively on one gender in special conditions, such as women killed in intimate partner relationships [16, 17].

In this study, homicides are examined regarding gender-specific characteristics to gain a comprehensive understanding of the methods and situations in which women and men are killed, including the law enforcement of these acts.

## Materials and methods

In a retrospective cross-sectional study, a file analysis was conducted of homicides from 2012 to the end of 2019 in which victims aged 14 years and older were autopsied at the Institute of Forensic Medicine in Mayence. The lower age limit was set at 14 years, since homicides of children are based on different problem situations compared to juveniles/ adults, which also requires an individual consideration of these offenses with regard to protective concepts. In addition, judicial information on further prosecution and, when possible, related court decisions were evaluated. Demographic data, information on the previous history with special emphasis on the perpetrator-victim relationship, aspects of the injuries and the type of violence, and influence of alcohol and drugs on the crime were recorded. Furthermore, aspects of law enforcement data and conviction of the perpetrators were evaluated. A gender-separated statistical evaluation was conducted, whereby the biological sex of the victims was differentiated. There were no transgender or intersex people among the victims in this collective.

Data were evaluated using the “IBM SPSS Statistics” software package (version 23). Frequencies, mean values, and medians were determined for descriptive analysis of the total collective. Furthermore, selected variables were evaluated by the chi-square test, respectively, the exact Fisher-test, to analyze significant gender differences in the offenses. A p-value of < 0.05 was considered statistically significant.

## Results

### Victims

**Fig. 1:**
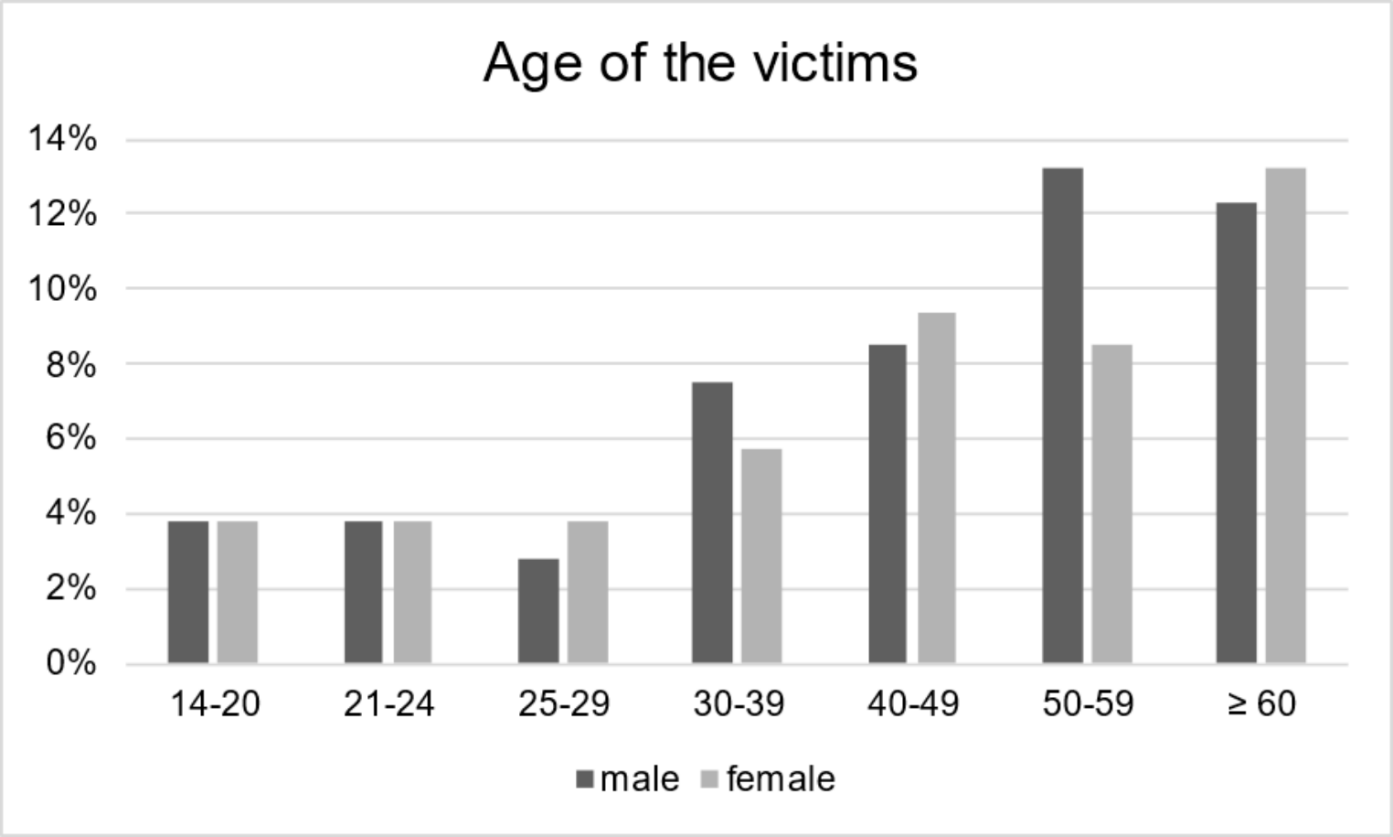
Age distribution of the victims

The Institute of Forensic Medicine autopsied 106 homicide victims between 2012 and 2019. The victims were 51.9% (*n* = 55) male and 48.1% (*n* = 51) female. On average, male victims were 48.2 ± 19.4 years (median 47 years), and female victims 48.5 ± 22 years (median 44 years) old (Fig. 1).

The victims were predominantly German (64.2%; *n* = 68), 34.9% (*n* = 37) were non-German citizens, and in 0.9% (*n* = 1) citizenship was unknown. Male victims were more often non-German than female victims (male 40.0%, *n* = 22; female 29.4%, *n* = 15), but the difference was not statistically significant.

### Perpetrators

The perpetrators were frequently male (84.0%) and between 30 and 49 years old (42.5%). Female perpetrators were less frequent (7.5%). In 8.5% the perpetrators could not be identified.

The perpetrators were predominantly German citizens (56.6%), 34.0% were non-German, one perpetrator (0.9%) held both German and non-German citizenship, and 8.5% of the perpetrators remained unidentified. Among male victims, the perpetrators were slightly more likely to be non-German than among female victims (male 38.2%; female 31.4%). The difference regarding the gender of the victims was not statistically significant.

In 37.3% of the female and 32.7% of the male victims, mental illnesses of the perpetrators were documented in the files. Difference regarding gender of the victims was not statistically significant. Addictive diseases of the perpetrators were reported statistically significant more often when the victim was male (49.1%) than female (27.5%), (*p* = 0.017) (Table 2).

**Table 2:** Statistically significant differences in female and male victims

	Male victims (*n* = 55)	Unknown	Female victims (*n* = 51)	Unknown	Significance
Perpetrator-victim relationship					
Perpetrator and victim known to each other	80.0%	12.7%	96.1%	3.9%	*p* = 0.039
Acquaintances/ friends	54.5	-	11.8%	-	*p* < 0,001 (Related to intimate partners and other acquaintances or relatives)
Intime partners	7.3	-	62.7%	-	
Family members	14.5	-	15.7%	-	
Other	3.6	-	5.9%	-	
Perpetrator unknown to the victim	7.3%	-	0.0%	-	
**Number of injuries**					
One to five injuries	52.7%	0.0%	25.5%	0.0%	*p* = 0.029
Six to fifty injuries	23.6%	0.0%	33.3%	0.0%	
**Crime scene**					
Victims’ home (or office)	36.4%	0.0%	76.5%	0.0%	*p* < 0.001
Other crime scenes	63.6%	0.0%	23.5%	0.0%	
**Substance influence of the victims (from criminal history)**	56.4%	10.9%	25.5%	11.8%	p = < 0.001
**Positive blood alcohol concentration of the victims**	40.0%	10.9%	17.6%	15.7%	*p* = 0.015
**Positive blood alcohol concentration of the perpetrators**	32.7%	43.6%	21.6%	27.5%	*p* = 0.019
**Addictive diseases of the perpetrators**	49.1%	18.2%	27.5%	19.6%	*p* = 0.017

In 39.2% of the female victims and 52.7% of the male victims, the perpetrators had prior convictions; however, in 24.5% of these cases, this information was absent. Differences regarding the gender of the victims were statistically not significant.

#### Previous assaults, verbal threats or stalking

In 55.7% of the total collective, a previous assault, verbal threat, or stalking was registered towards the victims. These events occurred more frequently among male (61.8%, *n* = 34) than female (49.0%, *n* = 25) victims, although the difference was not statistically significant.

#### Perpetrator-victim relationship

Female victims (96.1%, *n* = 49) were statistically significantly more likely to be killed by perpetrators they knew before than male victims (80.0%, *n* = 44), (*p* = 0.039). New acquaintances immediately before the crime were assigned to the category “unknown to each other”. In 3.8% (*n* = 4) of the total collective, perpetrator and victim were unknown to each other.

**Fig. 2:**
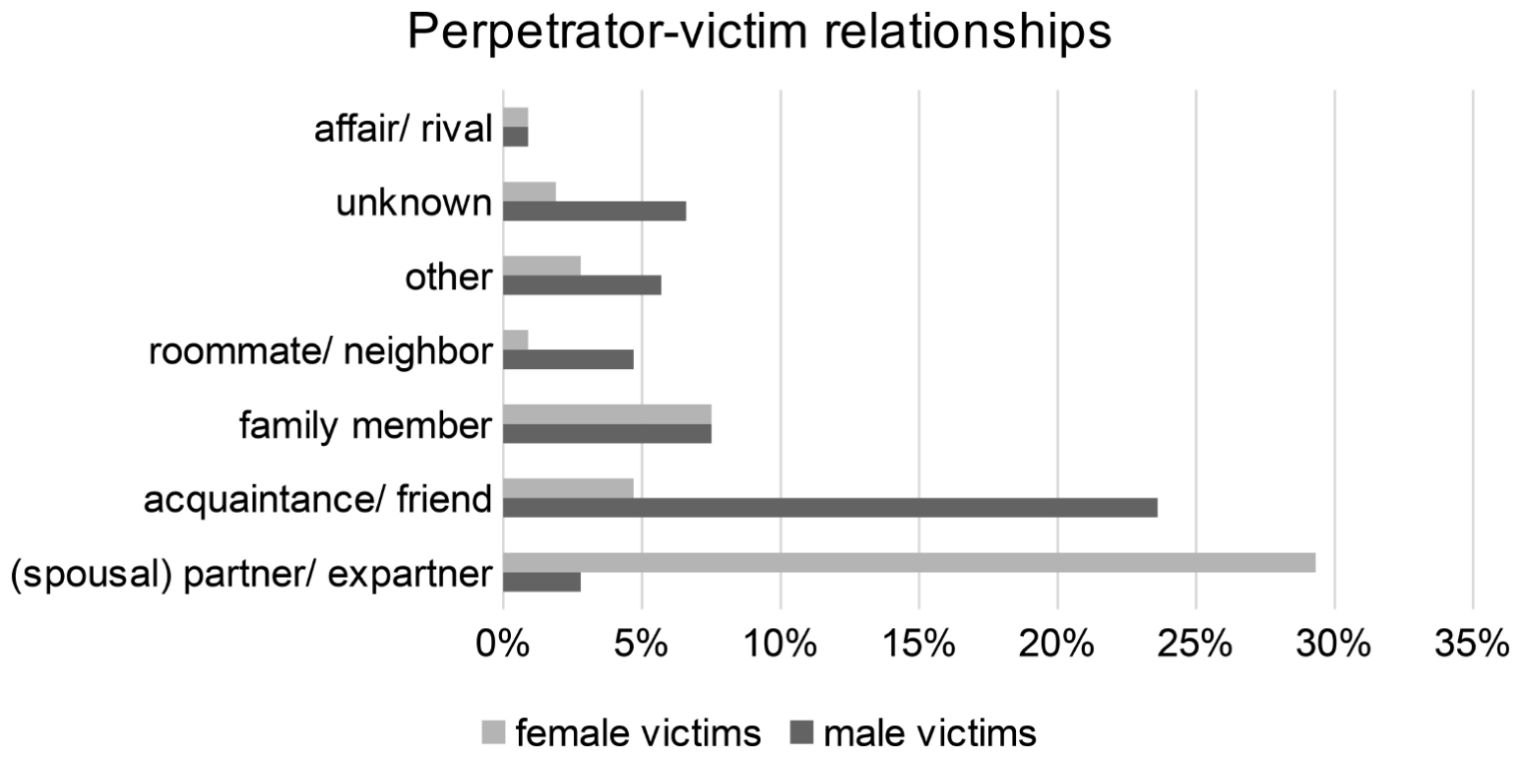
Perpetrator-victim relationships

In the total collective, the victims were killed in 34.0% each by acquaintances/ friends and intimate partners. Gender separated analysis showed, that male victims were most often killed by acquaintances/ friends (male 54.5%, *n* = 30) and female victims by intimate partners (62.7%, *n* = 32). In 15.1% (*n* = 16) of the total collective, the victims were killed by family members (without intimate partners), the gender distribution in these relationships was nearly equal (male 14.5%, *n* = 8; female 15.7%, *n* = 8). In the majority of these cases (*n* = 11), the perpetrators were adult (step)children (Table 2; Fig. 2).

To analyze statistically relevant differences in the perpetrator-victim relationships, the data were categorized into two groups: intimate partners and other acquaintances or relatives. The evaluation revealed that women were significantly more often killed by intimate partners, and men significantly more often by other acquaintances or relatives (*p* < 0,001).

#### Crime scene and time

**Fig. 3:**
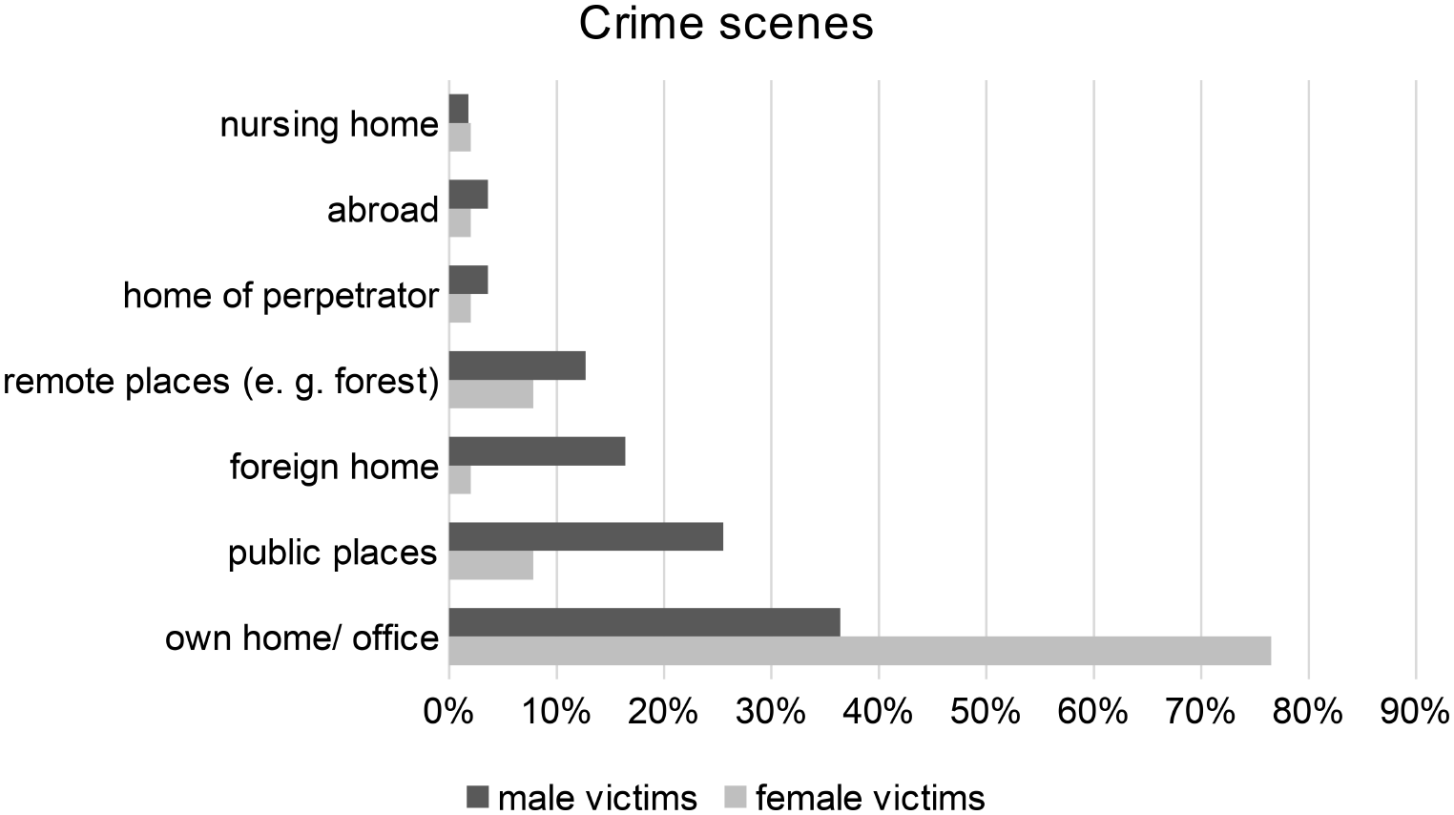
Crime scenes

Female victims (76.5%, *n* = 39) were killed twice as often as male victims (36.4%, *n* = 20) in their own home (including rarely own office) and male victims (63.6%, *n* = 35) nearly three times more likely (23.5%, *n* = 12) at other crime scenes (e.g. public places, a stranger´s home, forest or field). Difference regarding gender was significant (*p* < 0,001) (Table 2; Fig. 3).

Both male (54.5%, *n* = 30) and female victims (55.0%, *n* = 28) were killed primarily in the evening and at night (6 pm to 6 am).

#### Types of violence and instruments of crime

**Fig. 4:**
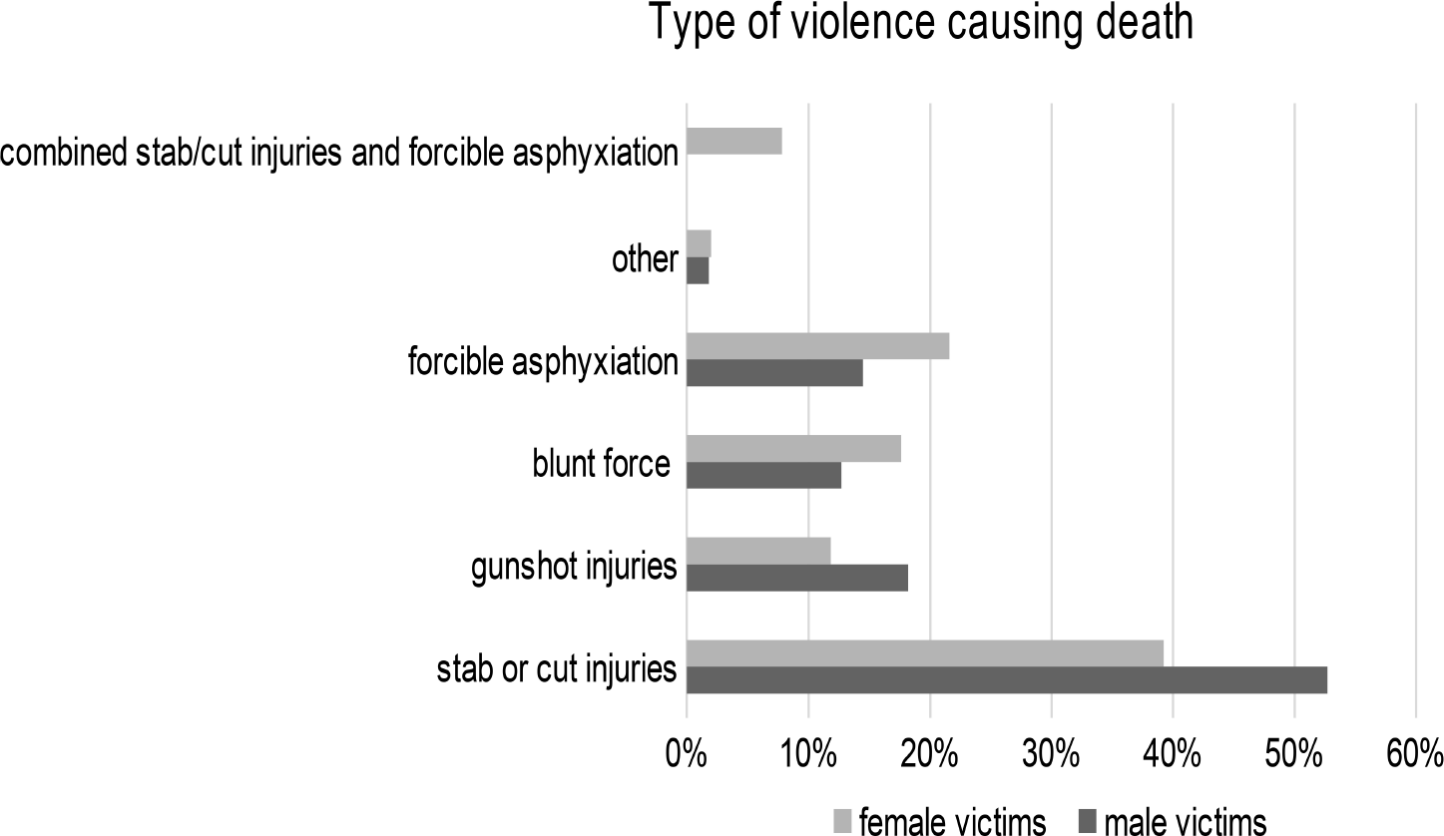
Type of violence causing death

Both male and female victims most frequently died from stabbing and cutting injuries, with men more likely to die from these injuries than women (male 52.7%, *n* = 29; female 39.2%, *n* = 20). Female victims were slightly more likely to suffer from forcible asphyxiation (male 14.5%, *n* = 8; female 21.6%, *n* = 11) and blunt force trauma (male 12.7%, *n* = 7; female 17.6%, *n* = 9). Gunshot injuries were more likely to occur to male than female victims (male 18.2%, *n* = 10; female 11.8%, *n* = 6) (Fig. 4).

Including injuries that were not the cause of death, in 67.9% (*n* = 72) of the total collective stabbing or shooting injuries were present. One to five injuries were found in 39.6% (*n* = 42) and six to fifty injuries in 28.3% (*n* = 30) of these cases. Male victims were significantly more likely to have one to five stabbing/ shooting injuries (male 52.7%, *n* = 29; female 25.5%, *n* = 13), while females were significantly more likely to have a variety (six to fifty) of these injuries (male 23.6%, *n* = 13; female 33.3%, *n* = 17); (*p* = 0.029). In nine cases with numerous injuries, partial or total exemption from criminal responsibility was assumed due to mental illness of the perpetrator.

**Fig. 5:**
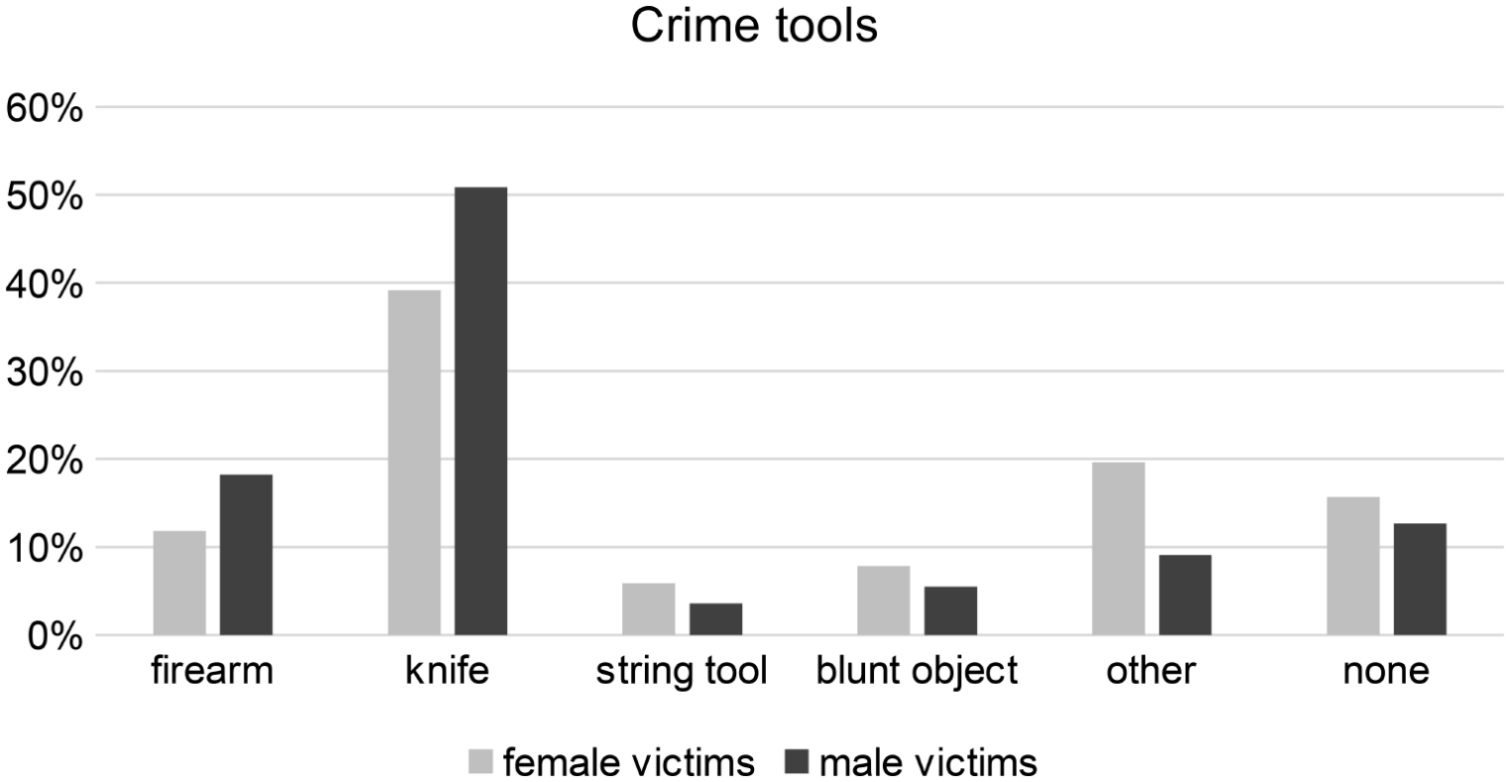
Instruments of crime

Instruments of crime were used in 85.8% of the cases, gender-separated analysis revealed no significant differences (female 84.3%, *n* = 43; male 87.3%, *n* = 48). The most frequently used instruments of crime were knives (45.3%, *n* = 48) and firearms (15.1%, *n* = 16), particularly among male victims (Fig. 5).

#### Substance influence of the victims

The criminal history indicates that 41.5% of the victims were influenced by alcohol, drugs, or medication at the time of the crime. In male victims the rate was significantly higher (56.4%, *n* = 31) than in female victims (25.5%, *n* = 13), (*p* = 0.001). The measure of blood alcohol concentration was positive in 40.0% (*n* = 22) of the male and 17.6% (*n* = 9) of the female victims (*p* = 0.015). In addition, toxicological analysis revealed substance influence by drugs and/or medication in 21.8% (*n* = 12) of the male and 15.7% (*n* = 8) of the female victims, although the difference was statistically not significant.

### Substance influence of the perpetrators

The case files revealed that alcohol, drugs, or medication influenced the perpetrators in 43.4% of the cases at the time of the crime. In these cases, the victims were more often male than female (49.1%, *n* = 27 vs. 37.3%; *n* = 19), but the difference was not statistically significant. In 32.7% (*n* = 18) of the male and 21.6% (*n* = 11) of the female victims, the blood alcohol measure of the perpetrators revealed positive results (*p* = 0.019) (Table 2). A toxicological analysis of the perpetrators revealed the influence of drugs or medication in 25.5% (*n* = 14) of the male victims and 19.6% (*n* = 10) of the female victims, but the difference was not statistically significant.

### Suicide of the perpetrator

The analysis revealed that 8.5% (*n* = 9) of the perpetrators, who were exclusively men, committed suicide immediately after the crime. These were almost exclusively partnership offenses with female victims (15.7%, *n* = 8).

### Exemption from criminal responsibility

In 13.2% (*n* = 14), a forensic psychiatric expert opinion determined a partial exemption from criminal responsibility (Sect. 21 StGB) and in 15.1% (*n* = 16) a total exemption from criminal responsibility (Sect. 20 StGB). In 24.5% (*n* = 26), no information was available. There were no significant differences regarding the gender of the victims.

### Conviction

24.5% (*n* = 26) of the final verdicts were ruled as manslaughter by the court, and 27.4% (*n* = 29) were ruled as murder. In 5.7% (*n* = 6), the court ruled bodily harm resulting in death. Robbery resulting in death (1.9%; *n* = 2) and intentional intoxication (cases in which the offender intentionally intoxicates themselve so that their criminal responsibility is limited or excluded at the time of the offense, section 323a StGB) (0.9%; *n* = 1) were rare verdicts, just in cases of male victims.

**Fig. 6:**
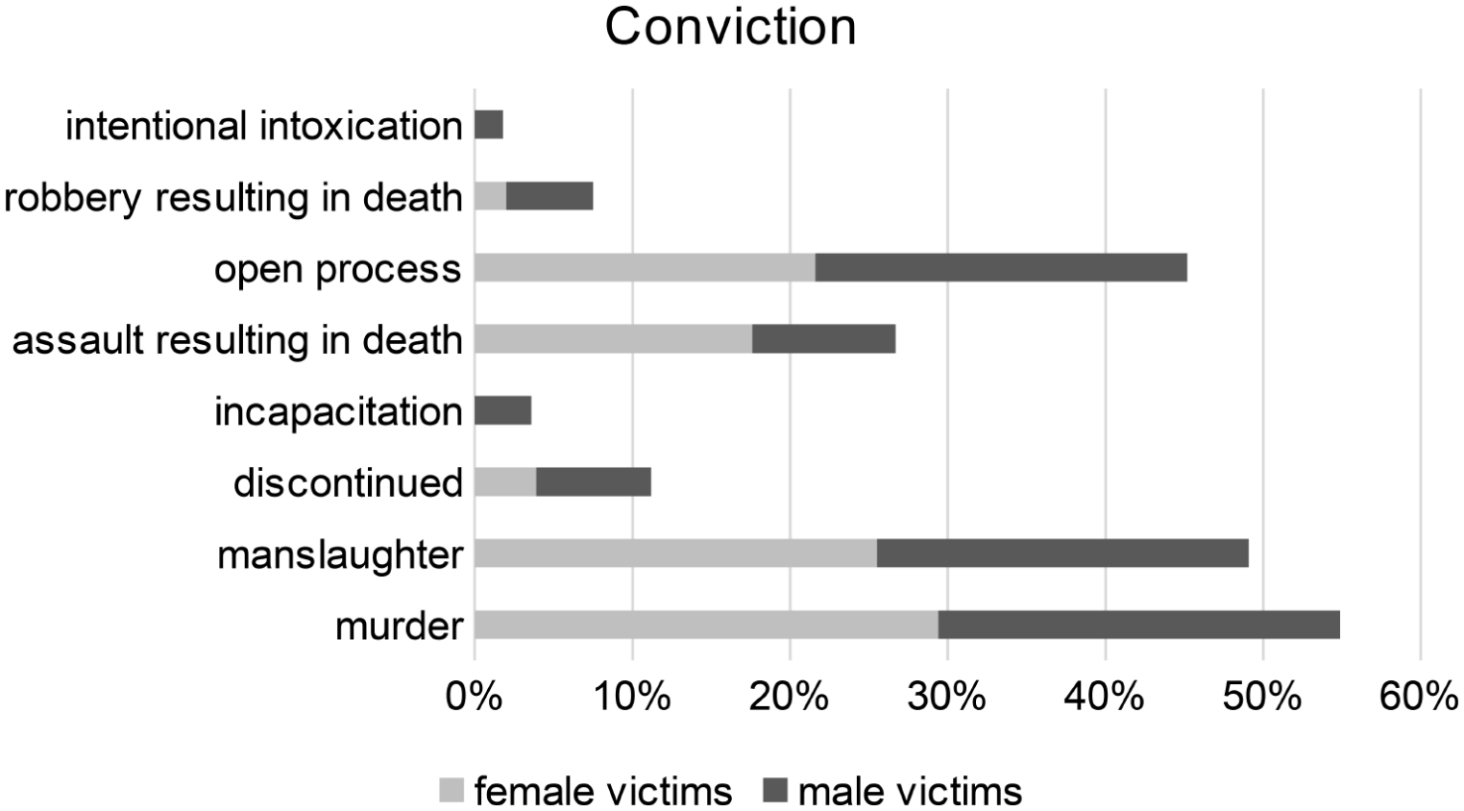
Conviction of the perpetrators

In 22.6% (*n* = 24), criminal cases were dismissed due to suicide of the perpetrator, unidentified or fugitive perpetrators, and total exemption from criminal responsibility. In 13.2% (*n* = 14) of the cases, murder, manslaughter, or bodily harm resulting in death was determined due to total or partial exemption from criminal responsibility, and placement of the perpetrators in a psychiatric hospital was ordered. In 3.8% (*n* = 4), criminal proceedings were still ongoing. There were no significant differences in the court decisions regarding male and female victims (Fig. 6).

## Discussion

The recording of homicide victims is not uniform, even across European countries. The same applies to differences in gender in the context of homicides. According to data from the United Nations, in European countries in 2012–2019 on average 72.3% male and 27.7% female victims of intentional homicide were registered [18]. Official data in Germany revealed an almost balanced gender distribution of the victims (2022: 46.4% male, 53.4% female; 2021: 49.9% male, 50.1% female) [3, 19], which is comparable to the results of this study.

Despite the nearly even distribution of male and female victims of homicide, this study found significant gender differences in the circumstances of these offenses and the relationships between victims and perpetrators. According to the results the perpetrators were most often male (84.0%), which is consistent with the literature (global rate > 90%) [4]. The number of homicides increased with the age of the perpetrators and victims (> 30 years). This age distribution is comparable with the figures from other European countries [4]. Globally, however, perpetrators and victims are most often between 15 and 29 years old, which has been linked to gang and organized crime prevalence, for example, in the United States [4].

The results show that men were often killed in public spaces (63.6%), while women were killed mainly in private spaces (76.5%). The crimes in both groups usually occured in the evening or at night (approx. 55%). To determine the proportion of foreign perpetrators and victims in this collective, comparisons with the general population are first necessary. In the catchment area of the Institute of Forensic Medicine in this study, the proportion of foreigners averaged 9.5% between 2012 and 2019 [20]. The perpetrators and victims in this collective were disproportionately often foreigners. The proportion of foreigners was also higher among male than among female victims (40.0% vs. 29.4%). In general, low-income population groups in unstable social circumstances are among the risk groups for violent crime [21]. Against this background, it should be mentioned that the social situation could in principle have a distorting influence when assessing the possible risk factor “origin” in homicides.

In the present collective male victims were killed most often by acquaintances or friends (54.5%). The homicides against men occurred significantly more often in connection with the victims’ influence on substances (56.4%), but in these cases the perpetrators were also often under the acute influence of alcohol or drugs (49.1%). In addition the perpetrators often suffered from addictive disorders (49.1%) or had a criminal record (52.7%). Measures of blood alcohol concentration and toxicological analysis could not confirm the substance use in every case; in particular, the examinations of the perpetrators resulted in a positive finding in fewer cases. On one hand, this could be caused by delayed or missing investigations after the crime, e.g. when the perpetrator was identified later. On the other hand research shows, that the perpetrators could have tended to blame the homicide on alcohol intoxication or drug use [22].

Female victims in the present collective knew the perpetrators significantly more often than male victims (96.1% vs. 80.0%). The likelihood of their deaths in their own homes was twice that of men (76.5% vs. 36.4%). These circumstances already give an indication that women are more often killed in close social relationships. Examining the perpetrator-victim relationships confirms this assumption. The female victims in the present collective were killed most often by intimate partners (62.7%), according to this, the victims of partnership crimes in this study were almost exclusively female (88.9%). It is known from other studies that homicide in intimate partner relationships is linked to patriarchy and structural inequalities between men and women that place men in a position of superiority and dominance over women [8, 23]. In addition, killings by (former) intimate partners are the most common and globally widespread manifestation of killings of women [24].

Due to the high number of unreported cases, it can be assumed that domestic violence is reported less frequently than violence that occurs outside the home. This may also be related to the fact that cases of women killed are less likely to have recorded offenders with criminal records, previous assaults, verbal threats, or stalking. In order to reduce the number of homicides among women, combating intimate partner violence and domestic violence plays an important role. Physicians are often the first point of contact after domestic violence. The challenge here is to identify violence-related injuries as such and to address the violence. In Germany, however, there is no obligation to file a criminal complaint; rather, the decision lies with the person concerned.

Gender specific differences in the homicides of adolescent and adult victims within the family (without intimate partner homicides) were not found in the present collective. Whether there are nonetheless killings based on the gender of victims in the context of family crimes (e.g., so-called “honor killings”) could be the subject of further evaluations.

All victims in the present study underwent forensic autopsies, resulting in a differentiated picture of the killing modalities. Sharp force trauma was the most common cause of death in male (52.7%) and female victims (39.2%) and knives were the most commonly used instruments of crime in these cases. The use of weapons like knives does not require special skills or resources, as most people have access to them at home [25]. Men were more likely than women to die from stab and cut or gunshot injuries, while women were slightly more likely to die from forcible asphyxiation and blunt force trauma, in line with the literature [26]. In female victims, the killing seems to be slightly more often performed with the perpetrator’s hands alone or blunt objects. This circumstance could be due to the generally different physical strengths of men and women, which could explain the somewhat increased use of firearms and knives in crimes among men.

At the same time, it is noticeable that female victims with stabbing, cutting, or gunshot wounds were more likely than male victims to suffer a variety of injuries (33.3% vs. 23.6%). Because male perpetrators are mostly physically superior, aspects of overkill must be discussed in these cases. The phenomenon of overkill describes a homicide in which the number of injuries inflicted significantly exceeds the number of fatal injuries. Considering that women in the present collective were predominantly killed in intimate partnerships, this type of perpetrator-victim relationship could be related to the phenomenon of overkill. Furthermore, perpetrators’ mental illnesses seem to have had an impact on the number of injuries inflicted in this study. In other studies, the phenomenon of overkill also tended to correlate with homicides related to mental disorders (within the schizophrenia spectrum) and domestic violence [27, 28].

In the present collective, one in six offenders who killed a woman, but only one in fifty offenders who killed a man, committed suicide after the crime; the offenders were all male. Notably, nearly 90% of the suicides involved partnership offenses with solely female victims. A homicide followed by suicide is a rare, but in partnership relations, it is also a common phenomenon in the literature [29, 30]. In intimate partner homicides, the perpetrator is more likely to commit suicide if the offenses were motivated by jealousy and illness than other motives [31]. Professional case management should consider the possibility of the perpetrator’s subsequent suicide, particularly in homicides against women in intimate relationships, and the potential consequences for the social environment, such as children living in the family. It is possible that the perpetrator’s death alone can bring justice for the bereaved. However, there may also be a lack of a sense of justice, as there is no conviction of the perpetrator with a corresponding punishment.

Approximately a quarter of the crimes were classified as murder (27.4%) and manslaughter (24.5%), with no significant differences between male and female victims. For instance, the offenders responsible for the homicide of a female victim were not convicted of murder more frequently. However, the German distinction between murder and manslaughter alone does not allow any direct conclusions to be drawn about gender-specific motives. Gender-specific characteristics only enter into the assessment of “base motives”, which can lead to a conviction for murder (Table 1). However, this does not mean that convictions for murder are always made on these grounds. It would therefore be worthwhile to evaluate the assessments that led to the distinction between murder and manslaughter in the verdicts. Furthermore, research could shed light on how the German justice system deals with gender-specific differences in relation to homicide through a separate evaluation of sentencing considerations. According to Sect. 46 (2) StGB, these factors must be taken into account when sentencing. As a result, the penalty must be higher if gender-specific motives were present. This could also be the subject of further investigation. The question of how to deal with offenders who are not or only partially responsible must also be addressed separately. If the law denies these perpetrators the capacity for insight and control, the assumption of gender-specific intentions must also be treated with caution.

The results of the present study underscore the need for protection concepts to combat homicides in Germany. In 2022, the Council of Europe criticized the lack of a national action plan in Germany to combat violence against women, as required by the Istanbul Convention [32]. It is easier to establish protective concepts to prevent homicides in the public sphere than in the private one, which is why homicides, particularly those against women, require special attention in society, law enforcement, and legislation. The specifics of killings of women in more domestic contexts require further analysis to implement protection concepts, also in light of the Istanbul Convention.

## Conclusion

The study revealed significant differences in homicides against men and women in terms of the crime’s commission, the surrounding circumstances, and the perpetrator-victim relationships. commission of the crime and the surrounding circumstances, as well as the perpetrator-victim relationships. In contrast to men, women are killed primarily in private intimate partner relationships. Homicides against men are more common in public spaces, where crime prevention concepts are easier to install. Targeted sensitization of police officers to the criteria of gender-specific killings along with instruments for risk analysis and identification of high-risk cases, is necessary, particularly in the case of homicides against women in light of the Istanbul Convention, to ultimately prevent such acts.

## Data Availability

Data sets generated during the current study are available from the corresponding author on reasonable request.
